# Heterogeneity of resistance mutations detectable by next-generation sequencing in TKI-treated lung adenocarcinoma

**DOI:** 10.18632/oncotarget.9931

**Published:** 2016-06-09

**Authors:** Deborah A. Belchis, Li-Hui Tseng, Thomas Gniadek, Lisa Haley, Parvez Lokhandwala, Peter Illei, Christopher D. Gocke, Patrick Forde, Julie Brahmer, Frederic B. Askin, James R. Eshleman, Ming-Tseh Lin

**Affiliations:** ^1^ Department of Pathology, Johns Hopkins University School of Medicine, Johns Hopkins Hospital, Baltimore, Maryland, USA; ^2^ Department of Medical Genetics, National Taiwan University Hospital, Taipei, Taiwan; ^3^ Department of Oncology, Johns Hopkins University School of Medicine, Johns Hopkins Hospital, Baltimore, Maryland, USA

**Keywords:** EGFR, PIK3CA, tyrosine kinase resistance, next-generation sequencing, lung cancer

## Abstract

*EGFR*-mutated lung adenocarcinomas routinely develop resistance to tyrosine kinase inhibitors (TKI). To better characterize the relative frequencies of the resistance mechanisms, we analyzed 48 *EGFR*-mutated TKI-resistant specimens from 41 patients. Next-generation sequencing of post-treatment specimens detected *EGFR* p.T790M in 31 (79%) of 39 patients, *PIK3CA* mutations in 10 (26%), *EGFR* p.S768_V769delinsIL in one, and *KRAS* p.G12C in one. Five *PIK3CA* mutations were outside of codons 542, 545, and 1047. Three of four pre-treatment specimens did not carry the *PIK3CA* mutation found in the post-treatment sample. Small cell carcinoma transformation was identified in four patients; none had p.T790M, including two where p.T790M was identified in the co-existing adenocarcinoma. In p.T790M-mutated specimens, the allele frequency was less than 5% in 24% of cases. p.T790M allele frequency was usually lower than that of the sensitizing mutation indicating that the resistance mutation was present either in a subset of cells or, if the sensitizing mutation was amplified, in a subset of the sensitizing alleles of a dominant clone. Eight patients had multiple resistance mutations, suggesting either multiple separate resistant clones or a single clone harboring multiple resistance mechanisms. *PIK3CA* mutations appear to be a more significant resistance mechanism than previously recognized.

## INTRODUCTION

The identification of targetable activating mutations in a subset of patients with adenocarcinoma of the lung has transformed therapy for patients with advanced disease. Approximately 10–15% of Caucasian patients and 30–40% of Asian patients with non-small cell lung cancers (NSCLCs) harbor activating mutations in the *epidermal growth factor receptor* (*EGFR*) gene [1–3]. Analysis of tumors for these mutations has become standard of care [1, 2, 4]. Treatment of patients with *EGFR*-mutated metastatic NSCLCs with gefitinib, erlotinib and afatinib, first-generation and second-generation EGFR tyrosine kinase inhibitors (TKIs), has improved response rates, time to progression, and overall survival [5]. Unfortunately, despite initial response to TKI therapy, acquired resistance develops after a median of approximately 10–13 months in almost all patients [1, 2, 4].

Several mechanisms for acquired resistance to TKI therapy have been reported [5]. These include additional mutations in *EGFR* (40–60%) [4, 6] and mutations in *PIK3CA* (5%) [7] and *BRAF* (1%) [8], amplification of *MET* (5–10%) [6, 9] and *ERBB2* (12%) [10], phenotypic transformation such as to small cell carcinoma (3–14%) [6, 7] and the epithelial to mesenchymal transition [5, 7]. The most common resistance mechanism is the secondary acquisition of an *EGFR* p.T790M mutation, present in approximately in 40% to 60% of resistant patients [6, 11]. Other uncommon acquired resistance *EGFR* mutations include p.D761Y, p.T854A and p.L747S [12–14]. It is now common clinical practice to select patients for third-generation TKI inhibitors, such as Rociletinib, Osimertinib (AZD9291) and HM61713, on the basis of p.T790M detection[5, 15, 16]. Recently, *EGFR* p.C797S mutation was found to be a novel mechanism of acquired resistance to third-generation TKIs [17–19].

Next-generation sequencing (NGS) is a powerful tool both to identify low-level mutations in cancers and to increase the accurate assessment of small biopsy specimens, as is common after relapse. Because of its high sensitivity, NGS may detect the emergence of a resistant subclone within the tumor, even when it comprises a few percent of the tumor cells analyzed. The identification of these mutants will determine therapeutic options. In this retrospective cohort analysis using a validated clinical NGS assay, we survey our experience with detection of acquired resistance mutations to TKI therapy in a panel of 7 genes [20, 21].

## RESULTS

### Positive control and negative control specimens

The peripheral blood negative control specimens showed no mutations in 115 runs; all mutations in the positive control specimens were detected over those runs. The observed mutant allele frequencies (MAFs) were highly consistent, demonstrating that NGS is quantitative and precise (Supplementary Table S1).

### Level of background noise of *EGFR* p.T790M (c.2369C > T) mutation in FFPE specimens

**Figure 1:**
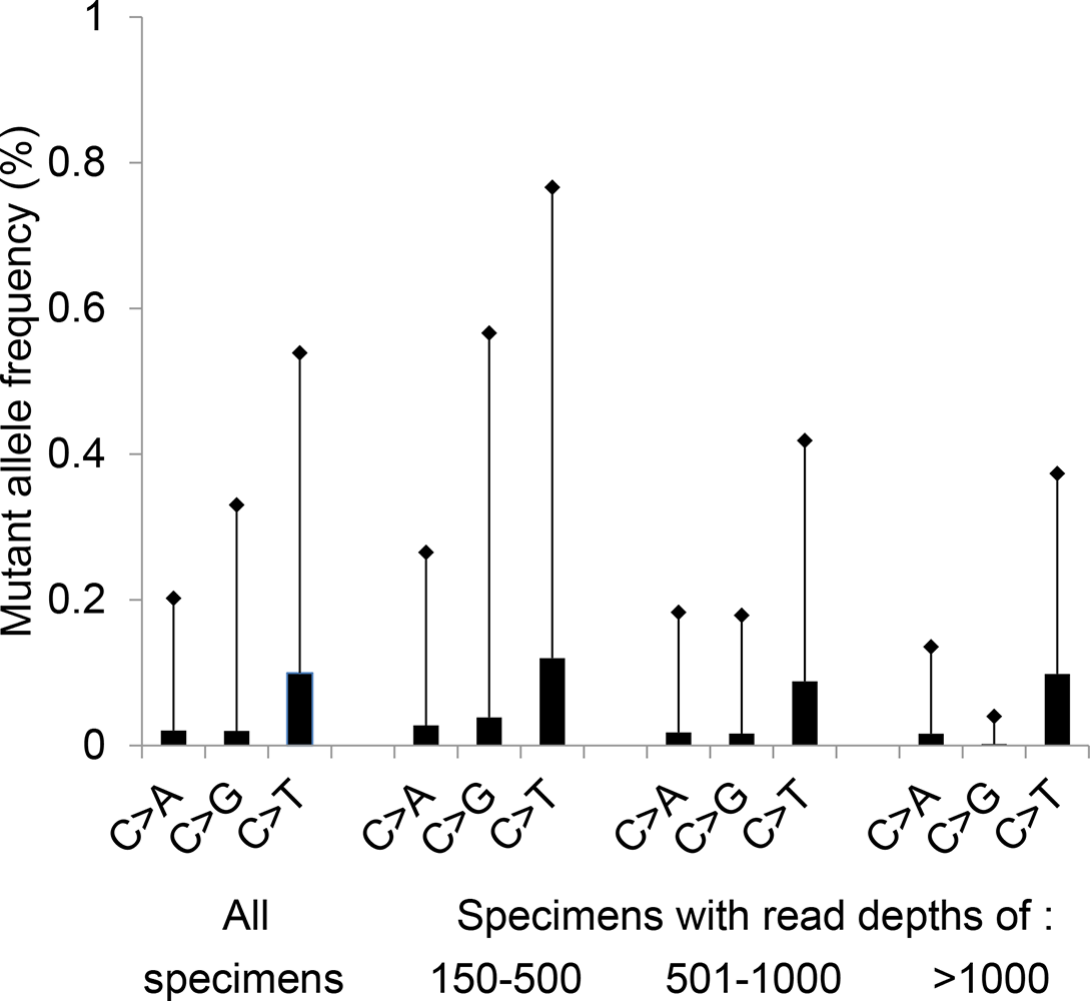
Background noise at *EGFR* c.2369C Mean plus 3 standard deviations (SD) of the variant frequency is plotted for a total of 179 specimens (Total); this includes 53 specimens with a read depth of 150-500 reads, 89 specimens with 501-1000 reads, and 37 specimens with more than 1,000 reads. All specimens contained an activating *KRAS* mutation at codons 12, 13, 61 or 146. The c.2369C > T change results in the p.T790M mutation.

In our previous clinical validation of this assay, the background noise for the *EGFR* c.2369C > T which results in p.T790M was calculated at 1.3% (mean plus 3 standard deviations (SD)), analyzing 16 FFPE non-neoplastic tissues [20]. For this study, a similar calculation of background noise for the *EGFR* c.2369C > T change was performed in 179 FFPE lung cancer specimens with an activating *KRAS* mutation. The C > T artifact (a deamination change) at position c.2369 was significantly higher than the C > A (*P* < 0.001) or C > G signal (*P* < 0.001) (Figure 1). The calculated background noise for c.2369C > T (mean plus 3 SD) decreased as read depth increased (0.77% for samples with 150-500 *EGFR* c.2369 reads, 0.42% for samples with 501–1,000 reads, and 0.37% for samples with more than 1,000 reads) (Figure 1).

### *EGFR* p.T790M mutation in pre-TKI specimens

Forty-one NSCLC patients who progressed after TKI treatment were included in this study. (Supplementary Table S2). *EGFR* mutations before treatment were examined at the Johns Hopkins hospital in 21 patients, 8 by Sanger sequencing and 13 by NGS. *EGFR* mutations were retrospectively analyzed in patients 3 and 4 whose EGFR mutations were initially tested by Sanger sequencing. Co-existing p.E746_A750del (68%) and p.T790M (7.6%) mutations were detected in patient 3. Other specimens with a MAF in c.2369C > T of 0.25% or less were interpreted as negative for p.T790M mutation.

### *EGFR* mutations in post-TKI specimens

Forty-eight post-TKI specimens were submitted from 41 patients whose NSCLCs progressed after TKI therapy. NGS failed in 5 of 48 specimens, including 2 from patients 16 and 20 who had only one specimen submitted for examination (Supplementary Table S2). TKI-sensitizing mutations were detected in all 39 patients, including 2 with an exon 18 mutation (p.G719C and p.E709_T710delinsD), 26 with an exon 19 deletion mutation, one with exon 20 p.A763_Y764insFQEA, and 10 with exon 21 p.L858R (Table 1). p.T790M was detected in 34 of 43 specimens or 31 (79%) of 39 patients (Tables 1 and 2), including specimen 2 with p.T790M at 1.1% MAF in 1059 reads. Retrospective analysis of 3 separate fragments from this pleural biopsy specimen showed relatively constant but low levels of mutation of p.T790M at 0.7% (1399 reads), 2.6% (1185 reads) and 2.6% (992 reads), respectively. Among the 6 additional *EGFR* mutations, 4 were present in both pre- and post-TKI specimens (patients 15, 17, 24 and 36), and one (p.S768_V769delinsIL or p.S768I plus p.V769L) was present in only the post-TKI specimen (Table 1). The type of *EGFR* mutation in the pre-TKI specimen was not known for patient 9, who had a p.K806I in the post-TKI specimen.

**Table 1:** Mutations and MAF in patients with TKI resistant tumors

Case^a^	*EGFR* TKI-sensitizing	*EGFR* T790M^b^	*PIK3CA*^c^^,^^d^^,^^e^	Other mutations^c^^,^^d^^,^^e^	mut no.^f^
1	E746_A750del (43%)	13%			1
2	L858R (17%)	1.1%^b^			1
3A	E746_A750del (57%)	16%	V344G (13%)^d^		2
3B	E746_A750del (39%)	13%	V344G (16%)^d^		2
3C	E746_A750del (66%)	27%	V344G (25%)^d^		2
4A	A763_Y764insFQEA (35%)	9.6%	G1049R (73%)^e^		2
4C	A763_Y764insFQEA (8.2%)	2.1%	G1049R (30%)^e^		2
5	E746_A750del (32%)	8.3%	E453K (16%)^c^		2
6	E746_A750del (26%)	5.8%			1
7	L858R (74%)	31%			1
8	E746_A750del (47%)	11%			1
9	L858R (8.6%)	6.1%	H1047L (6.9%)^c^	*EGFR*/K860I (8.5%)^c^	2
10	L747_A750delinsP (36%)	3.6%	E542K (6.1%)^d^	*EGFR*/S768_V769delinsIL (12%)^d^	3
11^a^	L858R (54%)	negative (432)			0
12	E746_A750del (52%)	22%			1
13	E746_A750del (68%)	negative (618)			0
14	L858R (11%)	negative (329)			0
15	E746_S752delinsIV(36%)	negative (291)		*EGFR*/G724S (47%)^e^	0
17	G719C (46%)	17%		*EGFR*/S768I (45%)^e^	1
18	L747_S752del (22%)	3.1%			1
19B	E746_A750del (9.2%)	2.0%			1
19C^a^	E746_A750del (64%)	negative (680)			0
21	E746_A750del (57%)	16%			1
22	E746_A750del (77%)	20%			1
23	E746_A750del (63%)	42%			1
24B	E746_T751delinsA (33%)	12%	Y1021C (16%)^c^	*EGFR*/K754Q (33%)^e^	2
25	E746_A750del (56%)	14%			1
26	E746_A750del (84%)	negative (494)			0
27	L858R (46%)	26%			1
28	L858R (34%)	4.3%	E110del (20%)^c^	*KRAS*/G12C (33%)^d^	3
29^a^	L747_P753delinsS (62%)	negative (2437)	E545K (23%)^c^		1
30	L747_T751delinP (32%)	14%	H1047R (21%)^c^		2
31	L747_P753delinsQ (29%)	10%			1
32	E746_A750del (65%)	33%			1
33	E746_A750del (47%)	4.1%			1
34	E746_S752delinsV (67%)	37%			1
35	L747_P755delinsSKG (9.4%)	negative (740)	E545K (8.3%)^d^		1
36	L858R (12%)	8.7%		EGFR/E709K (17%)^e^	1
37	L747_P753delinsS (91%)	5.0%			1
38	E709_T710delinsD (48%)	negative (2214)			0
39	E746_A750del (36%)	8.9%			1
40	L858R (43%)	13%			1
41	L858R (52%)	9.1%			1

a Small cell carcinoma. Others: adenocarcinoma.

b Number in parentheses indicates depth of coverage for specimens with negative p.T790M mutation. The depth of coverage was more than 150 reads for all p.T790M positive cases. Retrospective NGS assays of 3 subareas from the pleural biopsy of specimen 2 showed 0.7% (1399 reads), 2.6% (1185 reads) and 2.6% (992 reads) p.T790M, respectively.

c The presence or absence of mutation in pre-treatment specimens was not known.

d Mutations were not present in the pre-treatment specimens. Pre-treatment specimens of patients 3 and 4 were examined retrospectively by NGS.

e Mutations were present in the pre-treatment specimens.

f mut no.: The total number of resistance mutations in each patient's samples, including the *EGFR* 790M mutation, the *EGFR* p.S768_V769delinsIL mutation of specimen 10 (not present in the pre-treatment specimens), *PIK3CA* mutations, and the *KRAS* p.G12C mutation of specimen 28. The original TKI-sensitizing mutations and co-existing *EGFR* mutations in the pre-treatment specimens of patients 15, 17, 24 and the *EGFR* p.K860I mutation of specimen 9 were not included.

**Table 2:** *EGFR* p.T790M, *PIK3CA* and *KRAS* mutation in post-TKI specimens

	p.T790M	*PIK3CA*	*KRAS*	No mutation	2 or 3 mutations
Patients (*n* = 39)^a^	31 (79%)^b^	10 (26%)	1 (2.6%)	6 (15%)^e^	8 (21%)
adenocarcinoma (*n* = 37)	31 (84%)	9 (24%)	1 (2.7%)	5 (14%)	8 (22%)
small cell carcinoma (*n* = 4)^c^	0	2 (50%)^d^	0	2 (67%)	0

a Including patient 19 with an adenocarcinoma specimen and a small cell carcinoma specimen.

b including patient 2 with 1.1% p.T790M mutation.

c including retrospective analysis of the small cell carcinoma specimen in the pleural effusion of patient 4.

d The *PIK3CA* p.G1049R was present in the pre-TKI specimen of patient 4. The pre-TKI specimen of patient 29 was not tested.

e including patient 11 with small cell carcinoma transformation.

### p.T790M and TKI-sensitizing mutations are in a *cis* arrangement

**Figure 2:**
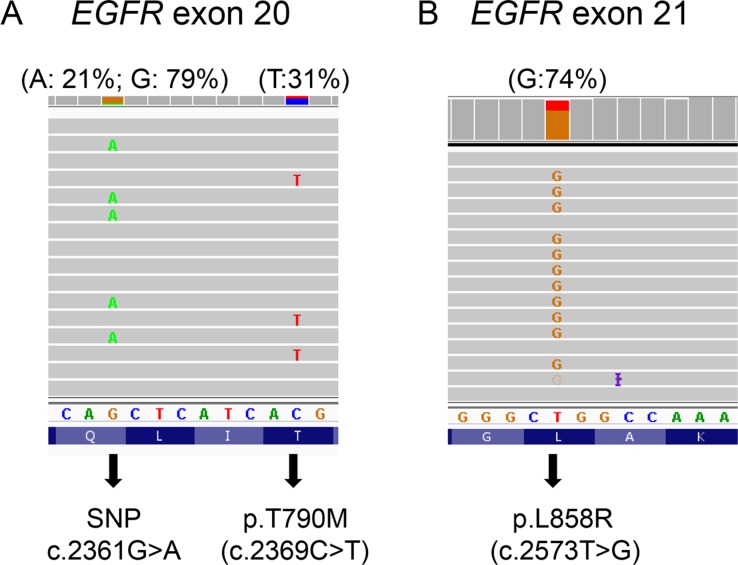
p.T790M in *cis* position of a subset of amplified p.L858R alleles In specimen 7 with an estimated 61–80% tumor cellularity, p.T790M (31%) was completely linked with a subset of SNP rs1050171 G allele, while p.L858R mutant allele frequency (74%) was similar to G allele frequency (79%). Percentage in the parentheses indicates mutant allele frequency.

By using amplicon-based NGS assays, the *cis* or *trans* relationship can be interpreted if co-existing variants are located within the same amplicon. p.T790M was located within the same allele with A763_Y764insFQEA in patient 4 and p.S768I in patient 17. The genotype of SNP rs1050171 was used to analyze 6 specimens in whom the variants were not on the same amplicon. By this method, a patient heterozygous for the inherited SNP will carry the initial, sensitizing mutation in linkage with only one of the SNP alleles (which are not on the same amplicon). If that chromosome is amplified, the SNP and sensitizing mutant alleles will be amplified together (> 50% allele frequency, similar in both SNP and mutant). A subsequent resistance mutation on the same amplicon with the SNP can then be imputed to be in either *cis* or *trans* with the sensitizing mutation. The MAF of the TKI-sensitizing mutation was always consistent with that of the SNP allele linked with p.T790M, indicating that p.T790M was in a *cis* configuration with the TKI-sensitizing mutant allele (Figure 2 and Supplementary Table S3). In specimen 10 with two acquired *EGFR* resistance mutations, p.S768_V769delinsIL was linked with the rs1050171 G allele, while p.T790M was linked with the A allele.

### *PIK3CA* and *KRAS* mutations in post-TKI specimens

**Figure 3:**
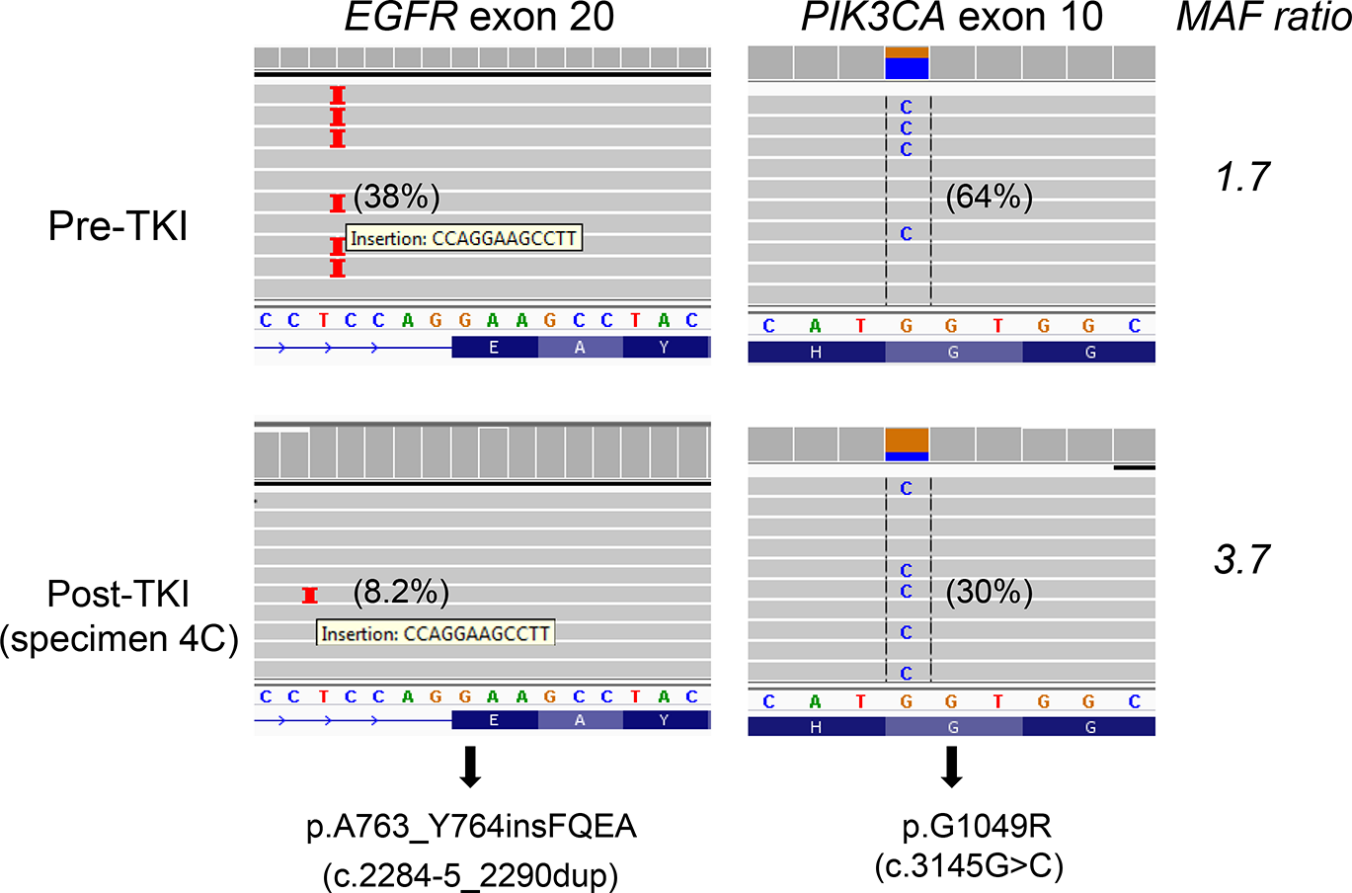
*PIK3CA* p.G1049R and *EGFR* p.A763_Y764insFQEA mutations in both pre-TKI (upper panel) and post-TKI (lower panel) specimens A 64% *PIK3CA* p.G1049R in the pre-TKI specimen (upper panel) suggests mutant allele-specific imbalance, which was confirmed by SNP array (Supplementary Figure S4). *PIK3CA*/*EGFR* p.A763_Y764insFQEA mutant allele ratio increased from 1.7 in the pre-TKI specimen to 2.1 (specimen 4A), 3.7 (specimen 4C) and 3.4 (pericardial effusion with small cell carcinoma) in post-TKI specimens. Repeated NGS showed *PIK3CA*/*EGFR* p.A763_Y764insFQEA mutant allele ratio was 1.6 in the pre-TKI specimen and 3.9 in post-TKI specimen 4C. Percentage in the parentheses indicates mutant allele frequency.

*PIK3CA* mutations were detected in 10 (26%) of 39 patients (Tables 1 and 2). The 3 most commonly reported codons (p.E542, p.E545 and p.H1047) for *PIK3CA* mutations accounted for 5 mutations. All except two with a *PIK3CA* p.E545K mutation (specimens 29 and 35) also carried an *EGFR* p.T790M. Four pre-TKI specimens were examined for the presence of the *PIK3CA* mutations known to be in their post-TKI specimens: p.G1049R was detected at both time points in patient 4 (Figure 3), but pre-TKI specimens were negative in patients 3 (Supplementary Figure S1), 10 and 35. In specimen 28, 4 mutations were detected (Table 1), including *KRAS* p.G12C (Supplementary Figure S2). The pre-treatment specimen of this patient was reported to be positive for *EGFR* p.L858R, but negative for *KRAS* mutation using the therascreen *KRAS* test at another CLIA-certified laboratory. No mutations were detected in the *AKT*, *BRAF*, *ERBB2* and *NRAS* genes in all post-TKI specimens examined.

### Small cell carcinoma transformation

Transformation from adenocarcinoma to small cell carcinoma was observed in 4 (9.8%) of 41 patients (Supplementary Table S2 and Table 2). The TKI-sensitizing mutation identified in the original pre-therapy adenocarcinoma was detected in the small cell carcinomas of all 4 patients; p.T790M was not identified in any. However, two (patients 4 and 9) of these patients had a component of adenocarcinoma remaining after TKI therapy, and each of these harbored p.T790M in the adenocarcinoma. *PIK3CA* p.G1049R, seen in the pre-TKI specimen, was also detected in the small cell carcinoma component of patient 4. *PIK3CA* p.E545K was observed in specimen 29, but the *PIK3CA* mutation status in the pre-TKI specimen was not examined.

### Mutant allele frequency of resistance mutations

**Figure 4:**
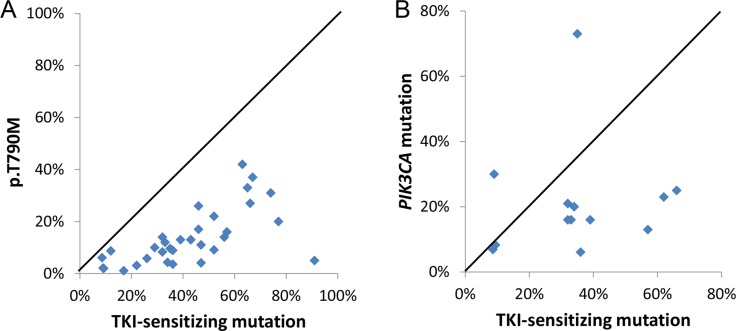
Higher TKI-sensitive *EGFR* mutant allele frequency than p.T790M mutant allele frequency (A) and *PIK3CA* mutant allele frequency (B)

p.T790M MAFs were 1–5% in 8 (24%), 1–10% in 16 (47%), and 1–20% in 27 (79%) of 34 specimens. In most post-TKI specimens, p.T790M MAFs were lower than the corresponding MAFs for sensitizing mutations (Figure 4A), indicating p.T790M is most likely present in a resistant subclone. However, since *EGFR* mutations in lung cancers are commonly associated with gene amplification [22], p.T790M could be present in all tumor cells but only on one or a subset of the amplified *EGFR* TKI-sensitive mutant alleles. In specimen 7, for example, the presence of 74% p.L858R indicates *EGFR* gene amplification, which was confirmed by a SNP array assay (Supplementary Figure S3A). The detection of 31% p.T790M in the context of 51–70% estimated tumor cellularity indicates that p.T790M was present in a subset of the amplified p.L858R mutant alleles within a dominant resistant clone (Figure 2). In support of this, the tumor was divided into five areas which were sequenced separately; the correlation of MAFs was analyzed by Spearman's rank correlation coefficient using GraphPad Prism software (GraphPad Software, ver5, La Jolla, CA) as described [23]. The sensitizing/resistance mutant ratio was constant (1.9, 2.1, 2.0, 2.1, 2.1; *r* = 0.71), arguing against a tumor subclone. Other examples included specimen 34 with 67% p.E746_S752delinsV and 37% p.T790M in the context of 71–90% tumor cellularity as well as specimen 3C with 66% p.E746_A750del and 27% p.T790M in the context of 71–90% tumor cellularity. *EGFR* gene amplification was also confirmed in these cases by SNP array assays (Supplementary Figure S3B and S3C)

*PIK3CA* MAFs were 1–10% in 3 (23%), and 1–20% in 8 (62%) of 13 *PIK3CA*-mutated specimens. As with p.T790M resistance mutations, *PIK3CA* MAFs in the post-TKI specimens were also equal to or lower than the TKI-sensitizing *EGFR* MAFs (Figure 4B), with the exception of two specimens (4A/4C). In this patient, a SNP array assay showed gain of the chromosomal region containing the *PIK3CA* gene in the pre-TKI specimen (Supplementary Figure S4). The *PIK3CA* p.A763_Y764insFQEA mutant allele ratio increased from 1.7 in the pre-TKI specimen to 2.1–3.7 in the post-TKI specimens (Figure 3).

### Co-existence of multiple of TKI resistance mutations

Two resistance mutations (*EGFR* p.T790M and *PIK3CA* mutations) were observed in 6 patients and 3 resistance mutations were observed in 2 patients (Tables 1 and 2). All 5 effusion specimens (3A, 3C, 4C, 9 and 10) from 4 patients showed 2 or 3 resistance mutations. Five specimens (13, 14, 15, 26 and 38) did not show mutations in *EGFR* p.790M, *PIK3CA* and *KRAS* genes nor transformation to small cell carcinoma; other proposed mechanisms of resistance (e.g., *MET* amplification) were not assessed.

## DISCUSSION

In contrast to prior studies, we identified a high frequency of *PIK3CA* mutations in post-therapy lung adenocarcinoma patients. At 26% (10 out of 39 patients), this is much higher than the frequency of 0–5% reported using a variety of non-NGS assay methods (Table 3)[6–8, 24]. Four of our cases with post-therapy *PIK3CA* mutations had pretreatment specimens available for analysis. Only one harbored a *PIK3CA* mutation, supporting our interpretation that these were acquired mutations. *PIK3CA* mutations are not common before treatment. In a multinational Lung Cancer Consortium study, *PIK3CA* was detected in approximately 1% of untreated cases [25]. Our unpublished results show a similar if slightly higher *PIK3CA* mutation rate (2.6%) in TKI-naïve specimens. Our elevated acquired *PIK3CA* mutation rate is most likely attributable to both higher analytic sensitivity and a broader reportable range of the NGS assay. Five of our *PIK3CA* mutations would not have been detected if the assay had been restricted to codons 542, 545 and 1047, the 3 most common spots for *PIK3CA* mutations. The MAF was less than 10% in 3 of the 5 mutations affecting codons 542, 545 and 1047. Overall, Sanger sequencing of exons 9 and 20 would have only detected p.G1049R (MAF: 73% and 30%), p.E545K (MAF: 23%) and p.H1047R (MAF: 21%) in patients 4, 29 and 30, respectively.

**Table 3:** Mutational profiling of TKI-resistant specimens

	*EGFR*	*AKT1*	*BRAF*	*ERBB2*	*KRAS*	*NRAS*	*PIK3CA*
Sequist et al. (*n* = 37)^a^	49%	ND	0%	ND	0%	0%	5%
Ohashi et al. (*n* = 146–212)^b^	55%	ND	1%	ND	0%	0%	ND
Yu et al. (*n* = 88)^c^	63%	0%	0%	0%	0%	0%	0%
Wu et al. (*n*= 20–42)^d^	48%	NA	0%	0%	0%	0%	0%
Current study (*n* = 39)	79%	0%	0%	0%	3%	0%	26%

a SNaPshot tumor genotyping assay to detect hot spots mutations of 13 genes, including 8 specific *PIK3CA* mutations within 6 codons [ref 7]. TKIs: gefitinib or erlotinib.

b A variety of methods including SNaPshot, mass spectrometry-based assay and Sanger sequencing to report mutations in the *EGFR* (*n* = 195), *BRAF* (*n* = 195), *KRAS* (*n* = 195), *MEK1* (146) and *NRAS* (*n* = 212) genes [ref 8].

c Mass spectrometry-based mutation profiling assay to identify 92 specific point mutation in 8 genes. Standard Sanger sequencing, fragment analysis and/or locked nucleic acid-based PCR sequencing were used to detected p.T790M mutation of 115 specimens [ref 6]. TKIs: gefitinib or erlotinib.

d Sanger sequencing of 10 genes included exons 9 and 20 of the *PIK3CA* gene [ref 23]. Specimens tested for *EGFR* were 42; other genes were tested in 20–26 specimens due to insufficient material. TKIs: afatinib with or without gefitinib or erlotinib.

In this study, 9 of the 10 post-therapy cases with *PIK3CA* mutations also possessed another resistance mechanism. Eight harbored a concomitant *EGFR* p.T790M mutation and the other case showed small cell carcinoma transformation. The finding of coexisting mechanisms of resistance has been described in studies examining post-TKI specimens [6, 26]. The significance of the *PIK3CA* mutation in these cases is uncertain. As *EGFR* acts through the *PIK3CA/AKT* pathway, mutations in that pathway might be anticipated to provide a resistance mechanism for the tumor cells. Interestingly, in the consortium study 2.7% of therapy-naïve specimens harbored multiple mutations [25]. Of these, *PIK3CA* mutation was a common target, found in association with another mutation in 48% of multiply mutated cases. A similar analysis of a broad variety of therapy-naïve cancers using a PCR-based mass spectrometry assay found that *PIK3CA* mutations are often associated with other driver mutations [27]. The significance of these mutations is uncertain. Several studies have suggested that its presence may affect outcome [28–30]. A group showed that EGFR-mutated NSCLC cell lines with acquired resistance due to either *EGFR* p.T790M mutation or *MET* amplification had increased apoptosis when a PIK3CA inhibitor plus a MEK pathway inhibitor were used simultaneously. Single agent PIK3CA inhibitor did not show increased apoptosis [31]. These pre-clinical findings may provide a rationale for co-targeting mutant EGFR and PIK3CA pathways to overcome resistance.

As with other studies, our analysis found the most common post-TKI mutation to be p.T790M, occurring in 79% of patients compared to a reported range of approximately 40% to 60%[6, 26]. Our higher percentage is most likely due to a higher analytic sensitivity of the NGS assay. Forty-seven percent and 79% of p.T790M-mutated specimens had MAFs less than 10% and 20%, respectively, which is below the limit of detection for Sanger sequencing. Twenty-four percent of p.T790M-mutated specimens had MAFs of 1–5% which is below the limit of detection for pyrosequencing, high resolution melting analysis or real-time PCR assays such as the therascreen EGFR RGQ PCR Kit and cobas EGFR Mutation Test [32–34]. In addition, these assays require separate runs for the detection of p.T790M and other *EGFR* mutations and therefore may not be suitable for comprehensive mutational profiling in core biopsy or fine needle aspiration specimens where tumor tissue is often limited. As shown in this study, many specimens were taken by core biopsy or fine needle aspiration.

The observation that p.T790M was present in only a small fraction of tumor cells of some patients implies the presence of additional resistant mechanisms in other subclones [35, 36]. In this study, we showed that p.T790M MAFs lower than TKI-sensitive MAFs did not always indicate that p.T790M was present in a small subpopulation of resistant tumor cells. Specimen 7 from our study shows that an acquired p.T790M was present in a fraction of amplified TKI-sensitive alleles of a dominant resistant clone and likely conferred TKI-resistance. Our observation is consistent with an *in vitro* study demonstrating the dominant effect of a low fraction of p.T790M alleles acquired in TKI-resistant cell lines with a high level of amplified TKI-sensitive alleles [37]. Such “allelic dilution” may obscure detection of the biologically significant *EGFR* resistance mutation [37] if assays with a lower analytic sensitivity are used. Correctly identifying the proportion of a resistant tumor containing p.T790M may be clinically important as it may correlate with the response to third-generation TKIs. Factors that could affect this analysis include sampling bias in choosing which portion of the tumor to test and allele amplification which might lead to inaccurate and/or imprecise estimation of tumor cellularity. Cell-free circulating tumor DNA has become an alternative source for non-invasive examination of p.T790M mutations [38, 39], with potentially less sampling bias as compared with tissue biopsy. Serial blood draws and ultra-sensitive quantitative assays, such as digital droplet PCR, may provide a precise measurement of p.T790M MAF in the cell-free circulating tumor DNA to correlate with treatment outcomes.

p.S768I has been categorized as a primary resistant *EGFR* mutation[40], although there are conflicting opinions from other studies with small case numbers [41, 42]. The largest cohort study showed TKIs were less effective in 7 patients with p.S768I mutation [43]. *EGFR* p.S768_V769delinsIL (or compound p.S768I and p.V769L) has also been detected in a pre-treatment specimen of a patient who did not respond to gefitinib [44], but has not been reported in TKI-resistant specimens. In our specimen 10, linkage of the two acquired mutations p.S768_V769delinsIL and p.T790M to the sensitizing exon 19 mutation could not be determined directly, but linkage of each with a different allele of SNP rs1050171 indicated that only one of these two mutants could be in *cis* with the TKI-sensitive mutant allele. A previous study showed a *cis* configuration between p.T790M and p.L8585R in two TKI-resistant cell-lines [37]. *In vitro* transfection also demonstrated greater TKI-resistance when the p.T790M allele was present in *cis* to the TKI-sensitizing mutation [37]. Further studies are warranted to elucidate if the p.S768_V769delinsIL confers TKI resistance and if an acquired resistance mutation is effective in the *trans* configuration with the TKI-sensitive mutant allele.

Mutations in *KRAS* and *EGFR* genes are mutually exclusive in therapy-naive NSCLCs, and *KRAS* mutations also appear to represent a negative predictor for TKI therapy in NSCLCs [45]. Surprisingly, acquired *KRAS* mutations were not detected in 4 previous larger cohort studies of re-biopsy tissues (Table 3) [6–8, 24], although co-existing *EGFR* p.L858R and p.T790M and *KRAS* p.G12V mutations were reported in a Chinese patient who progressed after 3 months of TKI therapy [46]. In our patient 28, we identified a post-therapy *KRAS*mutation that was not detected by a CLIA-certified laboratory using a relatively sensitive therascreen *KRAS* test; both pre- and post-therapy specimens showed an *EGFR* p.L858R mutation. To our knowledge, this is the first convincing example of a *KRAS* mutation acquired following TKI therapy, presumably as a resistance mechanism. The role of *KRAS* mutations in acquired TKI resistance was also supported by a recent study demonstrating a high incidence of *KRAS* mutations in the cell-free circulating tumor DNA from TKI-resistant NSCLC patients [47]. Further studies are needed to evaluate the analytic specificity (or background noise) of an assay with relatively high analytic sensitivity and to confirm the clinical specificity of an assay in circulating tumor DNA detecting a mutation commonly seen in other neoplasms.

In summary, in the setting of post-therapy NSCLCs NGS demonstrates an excellent limit of detection, a broad reportable range of mutations, the capacity for quantitative measurement of MAFs, and the ability to detect co-existing resistance mutations. MAFs can be used as a quality assessment measure to predict or identify heterogeneity of TKI-resistant tumors. Further studies are warranted to elucidate the clinical and/or biological significance of acquired *PIK3CA* mutations, *KRAS* mutations and uncommon *EGFR* (other than p.T790M) mutations as well as other co-existing resistance mutations in TKI-resistant lung cancers.

## MATERIALS AND METHODS

### Materials

Between April 2013 and June 2015, 830 formalin-fixed paraffin-embedded specimens with lung adenocarcinoma were submitted to the Molecular Diagnostics Laboratory at the Johns Hopkins Hospital. Forty-eight specimens were submitted from 41 patients whose NSCLCs progressed after gefitinib (one patient) or erlotinib (40 patients) therapy (Supplementary Table S2). Three specimens were submitted from patients 3, 4 and 19 and two specimens were submitted from patient 24. Duration of TKI therapy ranged from 3 months to 10 years with a median of 12 months. *EGFR* mutations before treatment were examined at the John Hopkins Hospital in 21 patients, 8 by Sanger sequencing of exons 18–21 and 13 by NGS [20]. In the remaining 20 patients the *EGFR* mutations before therapy were documented at other institutions. In 3 of these 20 (patients 9, 16, and 18) the details of *EGFR* mutations were not known. Thirty two were biopsy specimens, 7 fine needle aspiration specimens, 6 pleural effusion or ascites specimens, 2 resection specimens and one was a bronchioloalveolar lavage specimen (Supplementary Table S2). The Johns Hopkins Medicine institutional review board granted approval to this study

One hematoxylin & eosin (H&E) slide followed by 5–10 unstained slides, each with 5 or 10-micron thick sections, and one additional H&E slide were prepared with PCR precautions. The H&E slide was marked for tumor enrichment by a pathologist. Macro-dissection of neoplastic tissue from 3–10 unstained slides was performed. DNA was isolated using the Pinpoint DNA Isolation System (Zymo Research, Irvine, CA), followed by further purification via the QIAamp Mini Kit (Qiagen, Valencia, CA) [48].

### Next-generation sequencing (NGS)

NGS was conducted using the AmpliSeq Cancer Hotspot Panel (v2) for targeted multi-gene amplification, as described previously [20, 49]. Briefly, we used the Ion AmpliSeq Library Kit 2.0 for library preparation, Ion OneTouch 200 Template Kit v2 DL and Ion OneTouch Instrument for emulsion PCR and template preparation, and the Ion PGM 200 Sequencing Kit with the Ion 318 Chip and Personal Genome Machine (PGM) as the sequencing platform (Life Technologies, Carlsbad, California). The DNA input was up to 30 ng, as measured by Qubit 20 Fluorometer (Life Technologies). Up to 8 specimens were barcoded using Ion Xpress Barcode Adapters (Life Technologies) for each Ion 318 chip. One to three controls (a non-template control, a normal peripheral blood control from a male, and/or an artificial positive control specimen) were included on each chip. Positive controls were mixed DNA specimens from several cell lines with known mutations at low mutant allele frequency (lung cancer panel control): *AKT* p.E17K and p.E49K, *BRAF* p.V600E, *EGFR* p.T790M and p.L858R, *ERBB2* p.G776V or p.G776_V777insC, *KRAS* p.G12C and p.P121H, *NRAS* p.Q61R, and *PIK3CA* p.K111E and p.H1047R mutations (Supplementary Table S1).

Mutations were identified and annotated through both Torrent Variant Caller (Life Technologies) and direct visual inspection of the binary sequence alignment/map (BAM) file using the Broad Institute's Integrative Genomics Viewer (IGV). All specimens were analyzed for *AKT*, *BRAF*, *EGFR*, *ERBB2*, *KRAS*, *NRAS* and *PIK3CA* genes. During our validation of this NGS assay, a cutoff of background noise at 2% was chosen for single nucleotide variations because of our study of 16 non-neoplastic formalin-fixed paraffin-embedded tissues [20]. With sufficient DNA input, the limit of detection is dictated by the depth of coverage (or number of sequencing reads). Approximately 150 and 500 reads are needed to detect a heterozygous mutation with 99% confidence in a specimen with 20% and 10% tumor cellularity, respectively. The reportable ranges for the *AKT*, *ERBB2* and *EGFR* genes are summarized in Supplementary Table S4. Within these reportable ranges, rs1050171 (c.2361G > A, p.Q787 = of the *EGFR* gene) was the only single nucleotide polymorphism (SNP) with a minor allele frequency of more than 1% in the general population according to the dbSNP database of the National Center for Biotechnology Information. The reportable ranges and reference ranges for the *BRAF*, *KRAS*, *NRAS* and *PIK3CA* genes have been reported previously [21]. The nucleotide changes of the mutations detected in this study were summarized in Supplementary Table S5.

### Single nucleotide polymorphism (SNP) array

SNP array analysis was performed as previously described [50]. Briefly, DNA samples extracted from FFPE tissues (optimally 200 ng) were treated with the Infinium HD FFPE NDA restore kit before running on the Illumina Infinium II SNP array (HumanCytoSNP-12 v2.1 DNA Analysis BeadChip, Illumina Inc., San Diego, CA) according to the manufacturer's standard protocol. The B allele frequency and Log R ratio data were analyzed using Illumina KaryoStudio software version 2.0 and CNV (copy number variation) partition V2.4.4.0.

## SUPPLEMENTARY MATERIALS FIGURES AND TABLES


