# Supplementary material for: Heterogeneity of resistance mutations detectable by next-generation sequencing in TKI-treated lung adenocarcinoma
Source: Oncotarget. 2016 Jun 9;7(29):45237–48. doi: 10.18632/oncotarget.9931 (PMC5216719; doi:10.18632/oncotarget.9931)
Supplement: Supplementary file 1 [file oncotarget-07-45237-s001.pdf]

Heterogeneity of resistance mutations detectable by next-generation sequencing in TKI-treated lung adenocarcinoma

Supplementary Materials

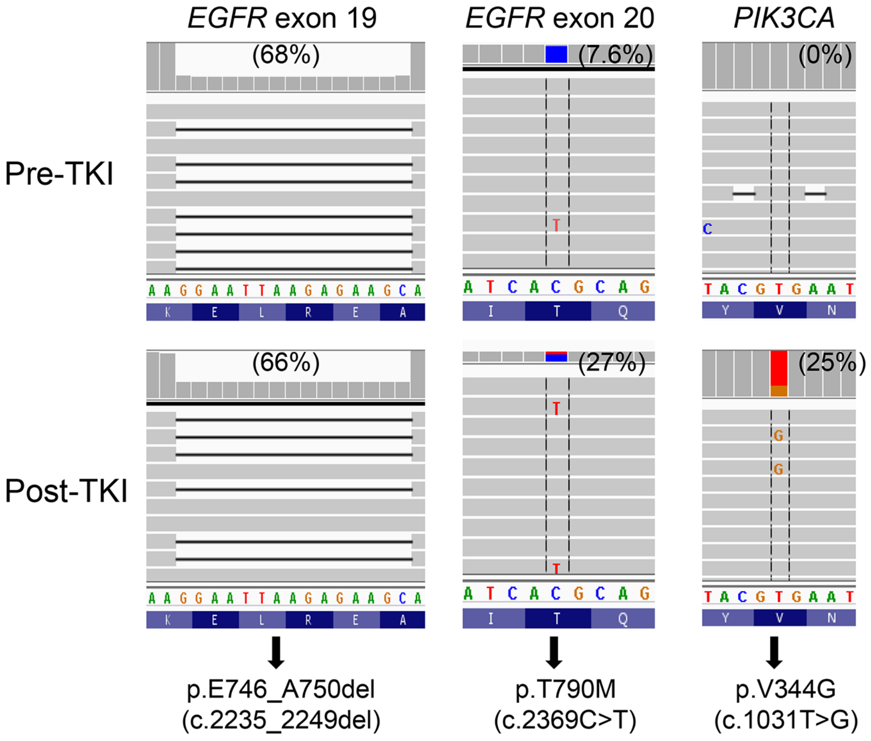

Supplementary Figure S1: NGS analysis of pre-TKI (upper panel) and post-TKI (lower panel) specimens of patient 3. *PIK3CA* p.V344G mutation was present only in the post-TKI specimen. Percentage in the parentheses indicates mutant allele frequency.

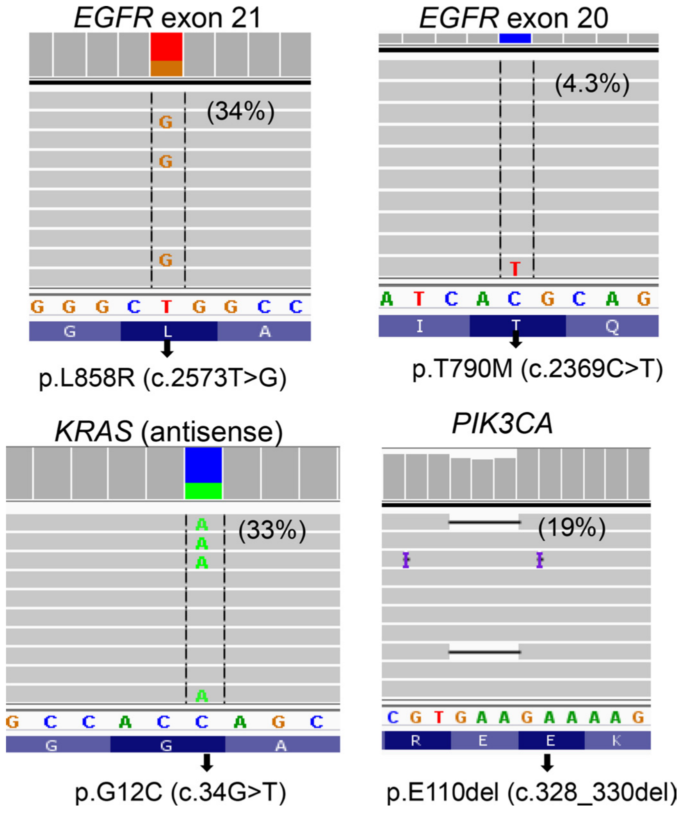

Supplementary Figure S2: NSG analysis of specimens 28. Percentage in the parentheses indicates mutant allele frequency (MAF).

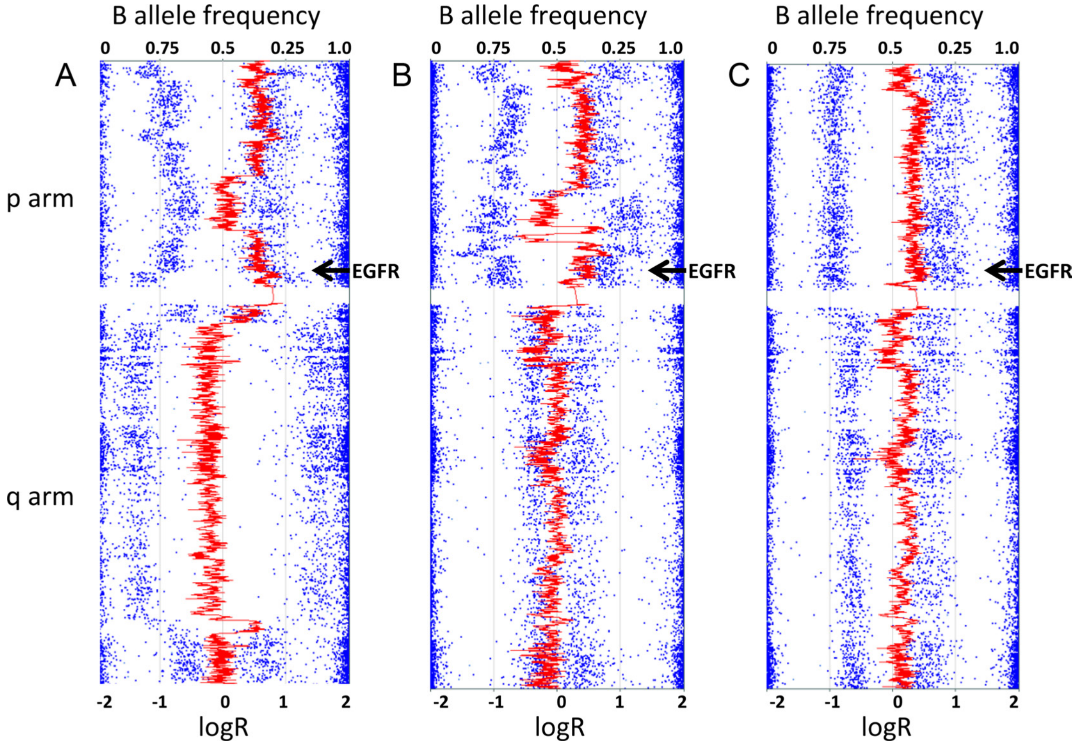

Supplementary Figure S3: SNP array analysis of specimens 7(A), 34(B) and 3(C). SNP array showed gain of chromosome 7p including the *EGFR* gene region.

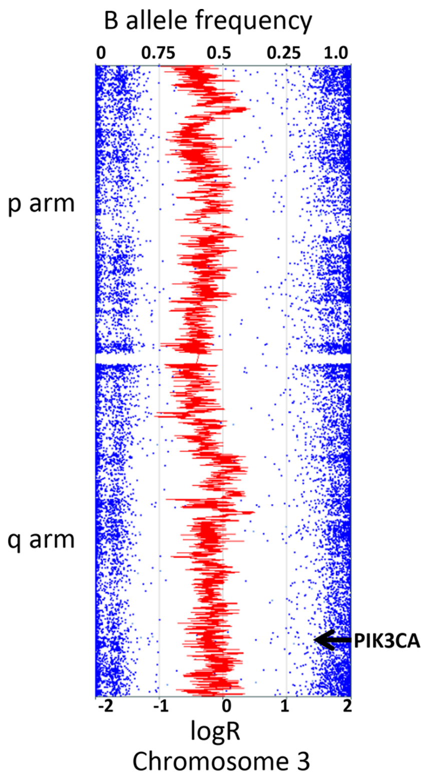

Supplementary Figure S4: SNP array analysis of the pre-TKI specimen of patient 4. SNP array showed copy neutral loss of heterozygosity (loss of the wild-type allele and duplication of the mutant allele) of chromosome 3q containing the *PIK3CA* gene.

Supplementary Table S1: Mutant allele frequency of lung cancer panel (LCP) controls[a]

Gene/Mutation (cell line)[b]	LCP control 1[c]	LCP control 2[c]	LCP control 3[c]
	(*n* = 61)	(*n* = 20)	(*n* = 31)
AKT/p.E17K (KU-19-19)	30 + 4.0%	24.2 + 3.0%	7.4 + 0.8%
AKT/p.E49K (KU-19-19)	32.9 + 2.6%	24.1 + 2.0%	8.8 + 1.3%
EGFR/p.T790M (NCI-1975)	30.1 + 1.9%	21.1 + 1.2%	9.0 + 1.5%
EGFR/p.L858R (NCI-1975)	21.9 + 3.1%	12.8 + 2.0%	5.8 + 0.9%
ERBB2/p.G776delinsVC (NCI-H1781)	NI	32.0 + 3.4%	NI
ERBB2/p.G776V (OVCAR-8)	6.8 + 0.6%	NI	8.5 + 0.9%

Abbreviations: n; number of times the control was run; NI: mutation not included.

[a]Results of the *BRAF*, *KRAS*, *NRAS* and *PIK3CA* mutations have been reported previously [20].

[b]p.E17K: c.49G > A; E49K: c.145G > A; p.T790: c.2369C > T; p.L858R: c.2573T > G; p.G776delinsVC: 2326_2327insTGT; p.G776V: c.2327G > T.

[c]mean ± standard deviation.

Supplementary Table S2: Forty-one patients with post-TKI specimens tested by NGS

Case[a]	Tissues	Pathology[b]	EGFR mutation before TKI[c]
1	lung/biopsy	adenocarcinoma	Exon 19 deletion (outside)
2	pleura/biopsy	adenocarcinoma	L858R (Sanger)
3A	right pleural effusion	adenocarcinoma	E746_A750del (Sanger)
3B	breast/biopsy	adenocarcinoma	
3C	left pleural effusion	adenocarcinoma	
4A	lung/FNA	adenocarcinoma[b]	A763_Y764insFQEA (Sanger)
4B[a]	lung/FNA	adenocarcinoma[b]	
4C	pleural effusion	adenocarcinoma[b]	
5	lung/biopsy	adenocarcinoma	E746_A750del (Sanger)
6	lung/resection	adenocarcinoma	E746_A750del (Sanger)
7	liver/biopsy	adenocarcinoma	L858R (Sanger)
8	lymph node/biopsy	adenocarcinoma	Exon 19 deletion (outside)
9	ascites	adenocarcinoma	EGFR (outside)
10	pleural effusion	adenocarcinoma	L747_A750delinsP (NGS)
11	soft tissue/biopsy	small cell carcinoma	L858R (Sanger)
12	lung/BAL	adenocarcinoma	E746_A750del (NGS)
13	liver/biopsy	adenocarcinoma	E746_A750del (outside)
14	lung/biopsy	adenocarcinoma	L858R (NGS)
15	lymph node/FNA	adenocarcinoma	E746_S752delinsIV and G724S (outside)
16[a]	liver/biopsy	adenocarcinoma	EGFR (outside)
17	liver/biopsy	adenocarcinoma	G719C and S768I (Sanger)
18	lymph node/biopsy	adenocarcinoma	EGFR (outside)
19A[a]	liver/FNA	small cell carcinoma	p.E746_A750del (NGS)
19B	pleural effusion	adenocarcinoma	
19C	liver/biopsy	small cell carcinoma	
20[a]	lung/biopsy	adenocarcinoma	L858R (NGS)
21	lymph node/biopsy	adenocarcinoma	E746_A750del (outside)
22	sacrum/biopsy	adenocarcinoma	E746_A750del (NGS)
23	lymph node/FNA	adenocarcinoma	E746_A750del (outside)
24A[a]	bone/resection	adenocarcinoma	E746_T751delinsA and K754Q (outside)
24B	chest wall/biopsy	adenocarcinoma	
25	lung/biopsy	adenocarcinoma	Exon 19 deletion (outside)
26	liver/biopsy	adenocarcinoma	E746_A750del (NGS)
27	liver/biopsy	adenocarcinoma	L858R (NGS)
28	lung/biopsy	adenocarcinoma	L858R (outside)
29	lymph node/FNA	small cell carcinoma	L747_P753delinsS (outside)
30	lung/biopsy	adenocarcinoma	L747_T751delinP (outside)
31	lung/biopsy	adenocarcinoma	L747_P753delinsQ (outside)
32	lung/biopsy	adenocarcinoma	E746_A750del (outside)
33	lung/biopsy	adenocarcinoma	E746_A750del (NGS)
34	lymph node/FNA	adenocarcinoma	E746_S752delinsV (NGS)
35	liver/biopsy	adenocarcinoma	L747_P755delinsSKG (NGS)
36	lung/biopsy	adenocarcinoma	L858R and E709K (NGS)
37	lung/biopsy	adenocarcinoma	L747_S753delinsS (NGS)

38	lung/biopsy	adenocarcinoma	E709_T710delinsD (outside)
39	lung/biopsy	adenocarcinoma	E746_A750del (outside)
40	pleura/biopsy	adenocarcinoma	L858R (outside)
41	lung/biopsy	adenocarcinoma	L858R (outside)

[a]Five specimens failed NGS testing.
[b]In patient 4, small cell carcinoma was present in the pericardial effusion and in left pleura and mediastinum autopsy specimens.
[c]Parentheses indicate source of sequence information. Outside, *EGFR* mutations were determined at other labs; NGS, Johns Hopkins NGS assay; Sanger, Johns Hopkins Sanger sequencing assay. The details of *EGFR* mutations were not known in patients 9, 16 and 18.

Supplementary Table S3: *cis* or *trans* relationship between TKI-sensitizing and p.T790M mutations[a]

Specimens	Mutant allele frequency		SNP allele linked with mutant allele[c]	
	TKI-sensitive mut	SNP (allele)[b]	TKI-sensitive mut	p.T790M
7	74%	79% (G)	G	G
21	57%	63% (G)	G	G
25	56%	63% (A)	A	A
34	67%	73% (A)	A	A
37	91%	90% (A)	A	A
41	52%	64% (A)	A	A

[a]The *cis* or *trans* relationship between TKI-sensitive and p.T790M mutations was deduced using genotyping of SNP rs1050171 (c.2361G > A) within exon 20 of the *EGFR* gene in 6 specimens with a 50% or more TKI-sensitive mutant allele frequency, presumably due to amplification of the *EGFR* gene.
[b]The nucleotide in the parenthesis indicates the genotype carrying the allele frequency.
[c]The SNP allele (A or G) linked with the TKI-sensitive mutant allele was predicted based on the concordant frequencies between TKI-sensitive mutant allele and SNP allele. The SNP allele linked with the p.T790M allele was observed based on the NGS analysis of an amplicon encompassing both SNP and p.T7900M positions (c.2361 and c.2369, respectively).

Supplementary Table S4: Reportable ranges of *AKT, ERBB2* and *EGFR* genes

Gene	cDNA position	Codon
AKT		
exon 3	47 to156	16 to 52
exon 6	459 to 547	153 to 183
ERBB2		
exon 19	2166 to 2217	722 to 739
exon 20	2218 to 2301	740 to 767
exon 21	2425 to 2556	809 to 852
EGFR		
exon 3	279 to 370	93 to 124
exon 7	835 to 889	279 to 297
exon 15	1723 to 1804	575 to 602
exon 18	2077 to 2179	693 to 727
exon 19	2185 to 2283	729 to 761
exon 20[a]	2284 to 2398 and 2406 to 2469	762 to 800 and 802 to 823
exon 21	2559 to 2625	853 to 875

[a]Two amplicons.

Supplementary Table S5: cDNA changes for the mutations documented in Table 1 and Supplementary Table S1

Case	*EGFR* (NM_005228)	*PIK3CA* (NM_006218)	*KRAS* (NM_033360)
1	E746_A750del (c.2236_2250del); T790M (c.2369C > T)		
2	L858R (c.2573T > G); T790M (c.2369C > T)		
3	E746_A750del (c.2235_2249del); T790M (c.2369C > T)	V344G (c.1031T > G)	
4	A763_Y764insFQEA (c.2284-5_2290dup); T790M (c.2369C > T)	G1049R (c.3145G > C)	
5	E746_A750del (c.2236_2250del); T790M (c.2369C > T)	E453K (c.1357G > A)	
6	E746_A750del (c.2235_2249del); T790M (c.2369C > T)		
7	L858R (c.2573T > G); T790M (c.2369C > T)		
8	E746_A750del (c.2235_2249del); T790M (c.2369C > T)		
9	L858R (c.2573T > G); K860I (c.2579A > T); T790M (c.2369C > T)	H1047L (c.3140A > T)	
10	L747_A750delinsP (c.2239_2248delinsC); S768_V769delinsIL (c.2303_2305delinsTTT); T790M (c.2369C > T)	E542K (c.1624G > A)	
11	L858R (c.2573T > G)		
12	E746_A750del (c.2235_2249del); T790M (c.2369C > T)		
13	E746_A750del (c.2235_2249del)		
14	L858R (c.2573T > G)		
15	E746_S752delinsV (c.2237_2255delinsT); G724S (c.2170G > A)		
16	EGFR (outside)		
17	G719C (c.2155G > T); S768I (c.2303G > T); T790M (c.2369C > T)		
18	L747_S752del (c.2239-2256del); T790M (c.2369C > T)		
19	E746_A750del (c.2235_2249del); T790M (c.2369C > T)		
20	L858R (c.2573T > G)		
21	E746_A750del (c.2235_2249del); T790M (c.2369C > T)		
22	E746_A750del (c.2236_2250del); T790M (c.2369C > T)		
23	E746_A750del (c.2236_2250del); T790M (c.2369C > T)		
24	E746_T751delinsA (c.2235_2251delinsAG); K754Q (c.2260A > C); T790M (c.2369C > T)	Y1021C (c.3062A > G)	
25	E746_A750del (c.2235_2249del); T790M (c.2369C > T)		
26	E746_A750del (c.2235_2249del)		
27	L858R (c.2573T > G); T790M (c.2369C > T)		
28	L858R (c.2573T > G); T790M (c.2369C > T)	E110del (c.328_330del)	G12C (c.34G > T)
29	L747_P753delinsS (c.2240_2257del)	E545K (c.1633G > A)	
30	L747_T751delinP (c.2239_2251delinsC); T790M (c.2369C > T)	H1047R (c.3140A > T)	
31	L747_P753delinsQ (c.2239_2259insCAA); T790M (c.2369C > T)		
32	E746_A750del (c.2236_2250del); T790M (c.2369C > T)		
33	E746_A750del (c.2235_2249del); T790M (c.2369C > T)		

34	E746_S752delinsV (c.2237_2255delinsT); T790M (c.2369C > T)		
35	L747_A755delinsSKG (c.2240_2264delinsCGAAAGG)	E545K (c.1633G > A)	
36	L858R (c.2573T > G); E709K (c.2125G > A); T790M (c.2369C > T)		
37	L747_P753delinsS (c.2240_2257del); T790M (c.2369C > T)		
38	E709_T710delinsD (c.2127_2129del)		
39	E746_A750del (c.2235_2249del); T790M (c.2369C > T)		
40	L858R (c.2573T > G); T790M (c.2369C > T)		
41	L858R (c.2573T > G); T790M (c.2369C > T)		
